# Polyene Macrolide Antifungal Drugs Trigger Interleukin-1β Secretion by Activating the NLRP3 Inflammasome

**DOI:** 10.1371/journal.pone.0019588

**Published:** 2011-05-23

**Authors:** Murthy Narayana Darisipudi, Ramanjaneyulu Allam, Khader Valli Rupanagudi, Hans-Joachim Anders

**Affiliations:** 1 Medizinische Poliklinik, University of Munich, Munich, Germany; 2 Department of Biochemistry, University of Lausanne, Epalinges, Switzerland; University Paris Sud, France

## Abstract

The use of antimycotic drugs in fungal infections is based on the concept that they suppress fungal growth by a direct killing effect. However, amphotericin and nystatin have been reported to also trigger interleukin-1β (IL-1β) secretion in monocytes but the molecular mechanism is unknown. Here we report that only the polyene macrolides amphotericin B, nystatin, and natamycin but none of the tested azole antimycotic drugs induce significant IL-1β secretion *in-vitro* in dendritic cells isolated from C57BL/6 mouse bone marrow. IL-1β release depended on Toll-like receptor-mediated induction of pro-IL-1β as well as the NLRP3 inflammasome, its adaptor ASC, and caspase-1 for enzymatic cleavage of pro-IL-1β into its mature form. All three drugs induced potassium efflux from the cells as a known mechanism for NLRP3 activation but the P2X7 receptor was not required for this process. Natamycin-induced IL-1β secretion also involved phagocytosis, as cathepsin activation as described for crystal-induced IL-1β release. Together, the polyene macrolides amphotericin B, nystatin, and natamycin trigger IL-1β secretion by causing potassium efflux from which activates the NLRP3-ASC-caspase-1. We conclude that beyond their effects on fungal growth, these antifungal drugs directly activate the host's innate immunity.

## Introduction

Innate immunity encompasses multiple strategies to impair the growth of fungi on external surfaces and in internal compartments of multicellular organisms. Innate pattern recognition receptors (PRRs) have the potential to detect and translate the recognition of fungal components into the transcription of NF-κB-dependent cytokines which triggers multiple aspects of host defense [1]. For example, fungal components like β-glucans and zymosans are the principle cell wall components of Candida, Aspergillus, S. cerevisiae and other fungi spp. are the potent pathogen associated molecular patterns to trigger different PRRs include toll-like receptors (TLRs) and C-type lectin receptors [1], [2]. The non-TLR PRRs, include dectin-1, mannose receptor, the Fcγ-coupled receptors Dectin2 and mincle, DC-SIGN, Galectin-3 and the scavenger receptors. The recognition and phagocytic internalization of fungal PAMPs by C-type lectins co-operates with TLR1, -2, -4 and -6 to activate MyD88 as well as spleen tyrosine kinase (SYK)/CARD9 signalling to produce numerous pro-inflammatory cytokines and chemokines.

Antimycotic drugs have dramatically improved the morbidity and mortality related to fungal infections. Antimycotics in clinical use encompass semisynthetic or fully synthetic compounds that have the capacity to kill fungi which substantially supports the host's immune system to eradicate the pathogen. As such antimycotic drugs and the host's immune defense act synergistically to control fungal infections. In this process dying fungi release additional agonists for pattern recognition receptors, therefore the early phase of antifungal therapy involves an additional activation of innate host defense. Interestingly, certain polyene antimycotic drugs like nystatin and amphotericin B have been reported to be able to directly induce interleukin-1beta (IL-1β) secretion in human peripheral blood mononuclear cells (PBMCs) and macrophages but the molecular mechanisms are still unknown [3], [5].

The activation of IL-1β secretion differs from that of other NF-κB-dependent cytokines as TLR- or C-type lectin signaling activates NF-κB to induce the expression of pro-IL-1β. In contrast to most other cytokines, the subsequent secretion of IL-1β requires a second signal, like inflammasome-mediated activation of caspase-1, also referred to as IL-1β converting enzyme [6]. Four inflammasomes have been described to integrate the various endogenous and exogenous triggers of caspase-1 activation, i.e. NLRP1, NLRP3, IPAF and AIM2 [6]–[9]. Of those, NLRP1 and NLRP3 were recently shown to be activated by microbial toxins. For example, Bacillus anthracis lethal toxin can activate caspase-1 via the NLRP1 inflammasome or pore forming toxins mitotoxin, vibrio toxins (V.cholerae, V.vulnificus) and bacterial ionophores nigericin, streptolysin O, and α, β and γ- hemolysins as well as muramyl dipeptides can activate NLRP3-mediated caspase-1 activation [10]–[12]. We therefore questioned whether synthetic antimycotic drugs have a similar potential to activate inflammasome- and caspase-1-mediated release of IL-1β as a mechanism to trigger innate immunity.

## Results

### Distinct polyene macrolide antifungal drugs activate dendritic cells and macrophages to secrete mature IL-1β

**Figure 1:**
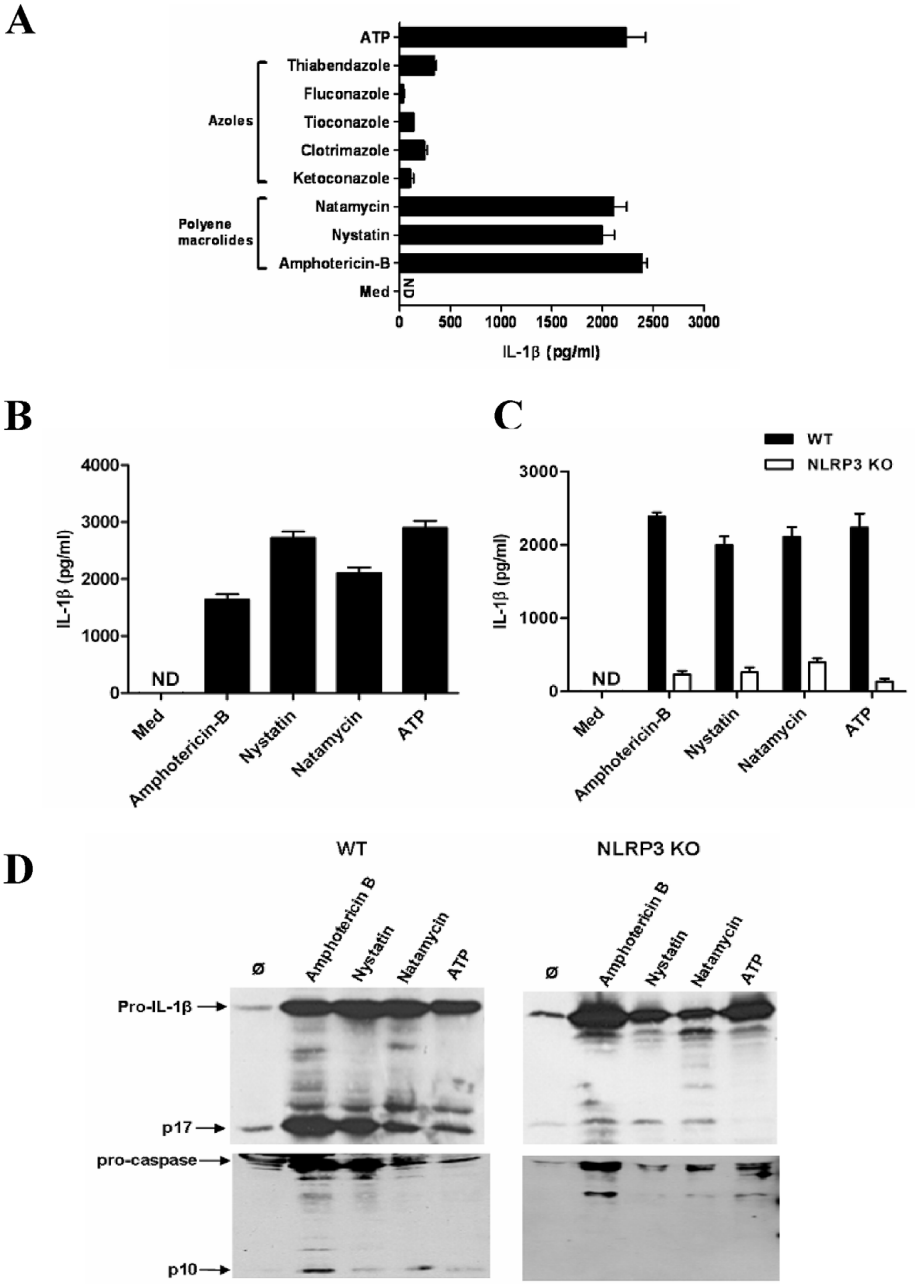
Amphotericin B, nystatin, and natamycin trigger IL-1β secretion via NLRP3. (A) LPS primed murine BMDCs and (B) macrophages were stimulated with antimycotic drugs as indicated at a concentration of 50 µg/ml; supernatants were collected 6 hours later, and IL-1β release was assessed by ELISA. ATP was used as a positive control. (C) LPS prestimulated wildtype or NLRP3-deficient BMDCs were exposed to amphotericin B, nystatin, and natamycin at a concentration of 50 µg/ml; supernatants were collected 6 hours later, and IL-1β release was assessed by ELISA. ATP was used as a positive control. (D) Immunoblot analysis of mature IL-1β (p17) and caspase-1 cleavage product (p10) in wildtype and NLRP3-deficient BMDCs stimulated with the drugs. Data are means ± SD from three independent experiments all performed in triplicate. p<0.05 versus medium.

To address a putative immunostimulatory potential, we first exposed LPS-primed bone marrow derived dendritic cells (BMDCs) to selected members of commonly used antifungal drugs and measured IL-1β production in cell culture supernatants after 6 hours of stimulation. Among all compounds tested only the polyene macrolides amphotericin B, natamycin, and nystatin induced high levels of IL-1β into BMDC supernatants (Figure 1A). Members of the azole antifungal drugs were far less potent to induce IL-1β secretion; therefore, we focussed on the polyene macrolides in the further experiments. This effect depended on priming with LPS (Figure S1) which provides the necessary signal for the induction of pro-IL-1β [6]. Similar results were obtained in BM-derived macrophages (Figure 1B).

### Amphotericin B, natamycin, and nystatin activate the NLRP3 inflammasome-ASC-caspase-1 to trigger IL-1β secretion

**Figure 2:**
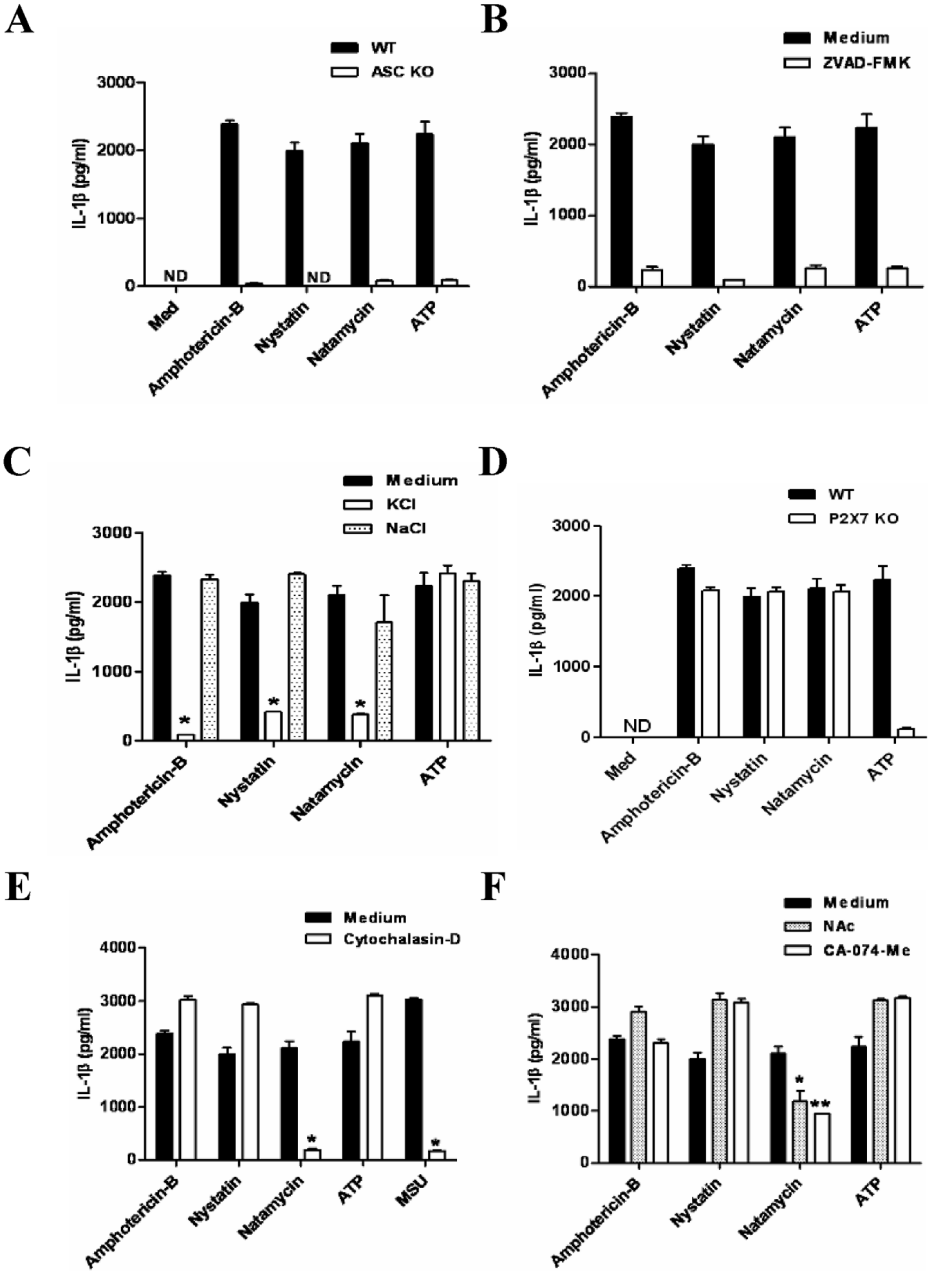
Mechanisms of amphotericin B-, nystatin-, and natamycin-induced NLRP3 activation. (A) IL-1β ELISA for wildtype and ASC-deficient BMDCs stimulated with the antimycotic drugs as indicated. Note that IL-1β secretion was dependent on the presence of NLRP3 and ASC for all drugs. (B) LPS prestimulated wildtype BMDCs were stimulated with the drugs in the presence or absence of the caspase inhibitor Z-VAD-FMK; supernatants were collected 6 hours later, and IL-1β release was assessed by ELISA. (C) LPS-primed wild type BMDCs were stimulated with antifungals in serum free buffer with or without 75 mM KCl or NaCl. IL-1β secretion was measured in supernatants after 6 hours (D). Wildtype or P2X7-deficient BMDCs were primed with LPS and exposed 50 µg/ml of each Amphotericin B, nystatin, and natamycin. IL-1β secretion was measured in supernatants after 6 hours of stimulation. ATP was used as control. (E) and (F) LPS-prestimulated wild type BMDCs were treated with cytochalasin D, NAC and the cathepsin inhibitor CA-07-Me for 30 min followed by stimulation with the antimycotic drugs for 6 hours. IL-1β secretion was measured in supernatants by ELISA. Data are means ± SD from three independent experiments all performed in triplicate. p<0.05 versus medium.

Various compounds such as crystals, pore-forming bacterial toxins or ATP induce IL-1β secretion by activating the NLRP3 inflammasome [13]. We therefore exposed amphotericin B, nystatin, and natamycin to BMDCs isolated from NLRP3-deficient or wildtype mice. Lack of NLRP3 almost completely abrogated IL-1β release upon stimulation with all three antifungals (Figure 1C). The remaining signal detected by IL-1β ELISA might represent extracellular pro-IL-1β. To address this possibility, we performed immunoblotting of cell culture supernatants to dissect the pro-IL-1β from the cleaved IL-1β (p17). In addition, immunoblotting for the p10 subunit of caspase-1 in cell culture supernatants of wildtype and NLRP3-deficient cells was performed to study caspase-1 activation. Stimulation with all three antifungals induced the caspase-1 cleaving product p10 in wildtype cells but not in supernatants of NLRP3-deficient cells, documenting that NLRP3 is required for drug-induced caspase-1 activation (Figure 1D). NLRP3 needs the adaptor ASC to activate caspase-1 [13]. In fact, lack of ASC as well as the pancaspase inhibitor Z-VAD-FMK abrogated IL-1β release in BMDC upon stimulation with all three antifungal drugs (Figure 2A and 2B). Thus, IL-1β secretion induced by amphotericin B, nystatin, and natamycin involves the NLRP3-ASC-caspase-1 pathway.

### Mechanism of NLRP3 inflammasome activation by polyene macrolide antifungal drugs

Several models of NLRP3 activation have been described, i.e. P2X7- and potassium flux-dependent pore formation [14], endosomal rupture involving cytosolic cathepsin B activity [15], and oxidative stress [16]. As some antifungals were shown to destabilize cell membranes and thereby promote potassium efflux [17]–[19], we first exposed BMDCs to medium containing 75mM potassium chloride which reduces potassium efflux. IL-1β secretion was abrogated upon stimulation with ATP as well as with amphotericin B, nystatin, and natamycin while a high extracellular concentration of NaCl had no effect (Figure 2C). To further confirm the effect of potassium efflux caused by these drugs in IL-1β induction, we treated cells with Quinidine, a potassium channel inhibitor. IL-1β secretion was significantly decreased with all drugs except for ATP (Figure S2). However, ATP-induced IL-1β secretion was different from that of the antifungal drugs because ATP-induced IL-1β secretion required the P2X7 receptor [14] while all three antifungal drugs could induce IL-1β release in P2X7-deficient BMDCs (Figure 2D), suggesting that all three drugs could directly trigger potassium efflux without activating P2X7, as most likely by introducing pores into the cell membrane [17]–[19].

The release of lysosomal proteases as a result of phagocytosis-dependent lysosome destabilization has also been implicated in NLRP3 activation [15]. In order to assess whether these antifungal drugs act through this pathway, we exposed BMDCs to all three antifungals in the presence or absence of the phagocytosis inhibitor cytochalasin D. Interestingly, cytochalasin D reduced IL-1β release only by natamycin, but did not influence IL-1β production by amphotericin and nystatin (Figure 2E). In addition, inhibition of cathepsin B by CA-074-Me, a specific inhibitor also produced the same results (Figure 2F), suggesting that only natamycin is involved in the lysosomal pathway to induce IL-1β production.

It has been shown that fungal ligands induce ROS and thereby activate NLRP3 inflammasome [16], [20], [21]. To determine the role of these drugs in NLRP3 activation, we treated LPS-prestimulated BMDCs with the antioxidant N-acetyl cysteine (NAc) followed by drug stimulation. Interestingly, NAc treatment significantly reduced the IL-1β production only by natamycin, but not by other drugs from the same group, suggesting that natamycin triggers IL-1β secretion in a different manner from that of amphothericn and nystatin.

Together Amphotericin B, nystatin, and natamycin activate the NLRP3 inflammasome through different ways but all are dependent on potassium efflux.

## Discussion

Our data show that polyene macrolide antimycotic drugs, namely amphotericin B, nystatin, and natamycin, activate the NLRP3-inflammsome- and caspase-1-mediated secretion of IL-1β in dendritic cells. IL-1β secretion will elicit secondary effects after IL-1R activation, hence, these antimycotic drugs have the potential to trigger innate immunity. This is remarkable in two ways: First, these compounds are known to inhibit fungal growth directly but our data show that they also might suppress fungal growth indirectly via activating innate host defense. Second, when used as an antifungal drug clinically, the immunostimulatory potential might contribute to the well known toxicity with these drugs. Azoles were far less potent as compared to polyene macrolides in inducing IL-1β secretion in dendritic cells, therefore, we focussed on this class of antimycotic drugs [3]–[5]. The polyene macroplids nystatin and amphotericin B had previously been reported to induce IL-1β relase in hPBMCs [5] and also in macrophages upon stimulation [22]. However, the mechanism how these drugs can trigger IL-1β release remained enigmatic.

IL-1β secretion requires two independent signals: First, the induction of pro-IL-β is a NF-κB-dependent process which requires the activation of TLRs, C-type lectins, TNFR or other signaling pathways leading to the degradation of IκB and the translocation of relA/relB to the nucleus [6]. Nystatin was previously reported to induce pro-inflammatory cytokines via TLR1/TLR2 signaling [3] but we found that nystatin, as well as amphotericin B and natamycin, required LPS prestimulation to induce pro-IL-1β as a prerequisite for IL-1β secretion. Hence, or data argue against a direct agonistic role of these drugs on TLRs.

The second step that is required for IL-1β secretion is the activation of caspase-1, an enzymatic step that cleaves off mature IL-1β from its pro-form. Our data clearly show that the NLRP3 inflammasome and ASC are necessary for polyene macrolides to activate caspase-1. NLRP3 is known to translate multiple stimuli into ASC-dependent caspase-1 activation [6]. So far three major danger signal pathways are known to convergate into the activation of NLRP3, i.e., potassium efflux through membrane pores, oxidative stress, and lysosomal destabilization with cytosolic cathepsin activity [6]. Our data clearly document that amphotericin B, nystatin, and natamycin all cause potassium efflux from dendritic cells, potentially by introducing membrane pores [17]–[19], which we found as a mandatory step for subsequent NLRP3 activation. This process did not involve P2X7 receptor which is consistent with previous descriptions of other NLRP3 agonists such as streptolysin O, hemolysins, and fungal components [20], [23]. However, the three antimycotic drugs differed in terms of the involvement of additional ways to activate NLRP3. Natamycin but not amphotericin B or nystatin involved the phagocytic uptake mechanims and cathepsin acitvity as it has been descfribed for various NLRP3-activating crystals [24]. Furthermore, ROS inhibition partially inhibited natamycin-induced IL-1β secretion as it has been described for high glucose, C. albicans, hemazoin and MSU crystals [13] as stimulators of intracellular oxidative stress [24].

Together, amphotericin B, nystatin, and natamycin have the potential to trigger IL-1β secretion in dendritic cells and macrophages by activating the NLRP3 inflammasome and ASC-mediated caspase-1 activation through potassium efflux. Thus, it is conceivable that these antimycotic drugs can directly activate innate immunity of the host conceptually similar to pathogen-associated molecular patterns.

## Methods

### Cell culture and reagents

Bone marrow-derived dendritic cells (BMDCs) and macrophages were isolated from 6 week old C57BL/6 mice (Jackson Labs, Bar Harbor, Maine) by established protocols. In some experiments BMDCs were prepared from NLRP3- [13], ASC- [8] or P2X7-deficient mice [25]. Cells were cultured in RPMI 1640 medium (Invitrogen, Carlsbad, CA, USA) supplemented with 10% FBS (v/v, Biochrom KG), 1% of penicillin and streptomycin (PAA Laboratories, Pasching, Austria). All cells were stimulated in serum free RPMI 1640 medium at a density of 1×10^6^ cells per ml. Cells were pre-stimulated with ultrapure LPS (50 ng/ml, Invivogen, San Diego, CA, USA) for 3hours and later cells were stimulated for 6 hours with antimycotic drugs (50 µg/ml, Sigma, Steinheim Germany), ATP (5mM) and MSU (250 µg/ml). Inhibitors such as Z-VAD-FMK (20 µM, all from Invivogen), cytochalasin D (5 µM), CA-074-Me (10 µM, Calbiochem, Darmstadt, Germany), NAC (10mM, Sigma) and Quinidine (250 µM, Sigma) were added 30min before antifungals stimulation. KCl (75 mM) was used to increase extracellular K+ concentration and NaCl (75mM) as a positive control.

### Measurement of cytokines

Mouse cytokines were measured in cell-free culture supernatants for IL-1β by ELISA from BD Biosciences Pharmingen (San Diego, CA, USA). Assays were performed in triplicate for each independent experiment.

### Immunoblotting

Cell-free culture supernatants were concentrated with Amicon ultra filters (Millipore, Billerica, MA) for standard immunoblot analysis as described [26]. Primary antibodies were polyclonal goat antibody to mouse-IL-1β (R&D Systems, Minneapolis, MN) and poly clonal rabbit antibody to anti-caspase-1 (sc-514, Santa Cruz, CA, USA).

### Statistical analysis

Data were expressed as mean ± standard deviation (SD). Comparison between two groups was performed by two-tailed *t*-test. A value of p<0.05 was considered to be statistically significant. All statistical analyses were calculated using GraphPad Prism.

## Supporting Information

Figure S1 **A. Antimycotic drugs-induced IL-1 β production depends on LPS prestimulation.** Wildtype BMDCs were primed with or without LPS (50ng/ml) for 3 hours. Cells were stimulated with 50 µg/mL concentration of each amphotericin B, nystatin, and natamycin. IL-1β secretion was measured in supernatants after 6 hours of stimulation. Data are means ± SD from three independent experiments all performed in triplicate. p<0.05 versus medium. **B. Antimycotic drugs-induced IL-1β production depends on Potassium efflux.** LPS (50ng/ml) primed BMDCs were treated with Quinidine (250 µM) for 30mins before cells were stimulated with 50 µg/mL concentration of each amphotericin B, nystatin, and natamycin. IL-1β secretion was measured in supernatants after 6 hours of stimulation. Data are means ± SD from three independent experiments all performed in triplicate. p<0.05 versus medium.
(TIF)
