# Supplementary figures and images for: Polyene Macrolide Antifungal Drugs Trigger Interleukin-1β Secretion by Activating the NLRP3 Inflammasome
Source: PLoS One. 2011 May 23;6(5):e19588. doi: 10.1371/journal.pone.0019588 (PMC3100296; doi:10.1371/journal.pone.0019588)

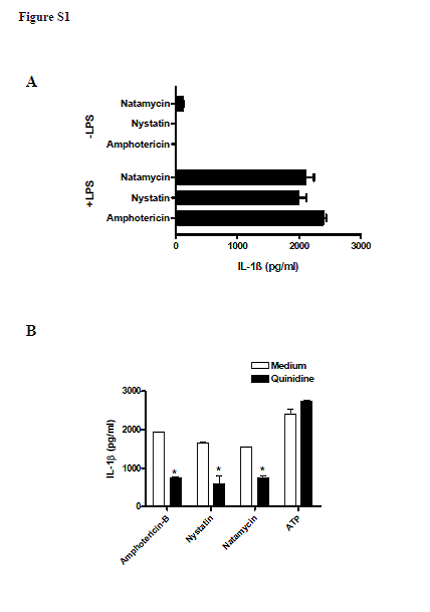

Supplement: Figure S1 — A. Antimycotic drugs-induced IL-1 β production depends on LPS prestimulation. Wildtype BMDCs were primed with or without LPS (50ng/ml) for 3 hours. Cells were stimulated with 50 µg/mL concentration of each amphotericin B, nystatin, and natamycin. IL-1β secretion was measured in supernatants after 6 hours of stimulation. Data are means ± SD from three independent experiments all performed in triplicate. p<0.05 versus medium. B. Antimycotic drugs-induced IL-1β production depends on Potassium efflux. LPS (50ng/ml) primed BMDCs were treated with Quinidine (250 µM) for 30mins before cells were stimulated with 50 µg/mL concentration of each amphotericin B, nystatin, and natamycin. IL-1β secretion was measured in supernatants after 6 hours of stimulation. Data are means ± SD from three independent experiments all performed in triplicate. p<0.05 versus medium. (TIF) [file pone.0019588.s001.tif]
